# Social support, health literacy and anxiety among pregnant women during coronavirus 2019 pandemic in Thailand

**DOI:** 10.3389/fpsyg.2023.1246996

**Published:** 2023-12-21

**Authors:** Piangkhuan Phutong, Suparp Thaithae

**Affiliations:** Department of Maternal – Newborn Nursing and Midwifery, Kuakarun Faculty of Nursing, Navamindradhiraj University, Bangkok, Thailand

**Keywords:** anxiety, pregnant woman, COVID-19, social support, health literacy

## Abstract

**Background:**

The ongoing coronavirus disease 2019 (COVID-19) pandemic continues to have a significant impact. Pregnant women are particularly vulnerable to its effects, which may increase their anxiety levels. This study aims to investigate anxiety levels in pregnant women during the COVID-19 pandemic in Thailand and to identify factors predicting such anxiety.

**Methods:**

The researchers collected data through an online questionnaire from November 2021 to May 2022. The sample included 404 pregnant women. The questionnaire consisted of personal information, health literacy related to COVID-19, social support, and anxiety related to COVID-19. The content validity of the questionnaire were verified by three experts, with content validity indices of 0.87, 0.80, and 0.87 for each domain, respectively. The reliability of the questionnaire were 0.96 for health literacy, 0.95 for social support, and 0.96 for anxiety. Moreover, in-depth telephone interviews were also conducted with pregnant women. The data were analyzed using descriptive statistics, stepwise multiple regression, and content analysis.

**Results:**

Group of 404 pregnant women were studied, and the results showed that pregnant women had a high level of health literacy regarding COVID-19 and pregnancy (mean = 96.36, SD = 14.23) and social support level on a high level (mean = 83.99, SD = 11.34). Most of them were concerned about anxiety related to COVID-19 infection and pregnancy on a moderate level (mean = 47.78, SD = 11.49). The factors predicting the anxiety of pregnant women during the COVID-19 outbreak in Thailand included health literacy related to COVID-19 (β = 0.468) and social support (*β* = 0.283), with a prediction rate of 32.80% (*R*^2^ = 0.328) with statistical significance (*p* < 0.05).

**Conclusion:**

This study revealed the anxiety level of pregnant women during the COVID-19 outbreak in Thailand, which was moderate. Health literacy about COVID-19 and social support can predict the anxiety level of pregnant women.

## Introduction

Coronavirus Disease 2019 (COVID-19) is an infectious disease caused by the SARS-CoV-2 virus. The initial case emerged on December 1, 2020, in Wuhan, the capital of Hubei province, China. Swiftly escalating, the virus proliferated globally, resulting in a surge of infected individuals and casualties. The World Health Organization (WHO) declared the coronavirus 2019 a global pandemic on March 11, 2021 (Cucinotta and Vanelli, 2020; Ansariniya et al., 2021). The most significant symptom is sudden respiratory failure, which can lead to difficulty breathing or pneumonia. However, most cases exhibit mild to moderate symptoms, such as fever, runny nose, cough, sore throat, headache, body aches, fatigue, loss of smell, nausea, vomiting, or diarrhea (Boddington et al., 2021). Severe symptoms occur in elderly patients and those with underlying medical conditions, such as diabetes, heart disease, respiratory disease, cancer, and pregnant women (Centers for Disease Control and Prevention, 2021). Although the risk of infection in pregnant women is similar to that of the general population, there is a higher chance of severe symptoms compared to non-pregnant women. The rapid progression and failure of the respiratory system may be due to changes in physiology and alterations in the immune system during pregnancy.

Aside from the impact on the health of pregnant women, COVID-19 infection also affects pregnancy outcomes. It leads to preterm birth at a rate of 19–49%, premature rupture of membranes at a rate of 19%, and fetal distress due to hypoxia at a rate of 43% (Almeida et al., 2020). Moreover, there is an increased incidence of stillbirths during the pandemic, from 2.38 per 1,000 births to 9.31 per 1,000 births (Khalil et al., 2020). Most of the research, around 70%, indicates that neonates contract COVID-19 from the environment after delivery, but transmission from mother to fetus during pregnancy is also possible (Centeno-Tablante et al., 2021). The World Health Organization (2020) recommends that mothers can continue to breastfeed their infants, with appropriate precautions and guidance from healthcare professionals on proper practices and the associated risks and benefits. Neonates with COVID-19 exhibit similar symptoms to adults, including fever (44%), gastrointestinal abnormalities (36%), respiratory abnormalities (52%), pulmonary abnormalities (64%), and neurological symptoms (18%) (Raschetti et al., 2020).

During the outbreak of COVID-19 in Thailand, the Ministry of Public Health implemented a policy to modify prenatal care services by reducing the duration of care and limiting certain activities such as parent school and reducing the number of prenatal visits to hospitals to minimize the risk of exposure to the virus (Ministry of Public Health, 2020b). Previous studies show that fetal wellbeing and daily lives restrictions are the greastest concern for pregnant women (Akgor et al., 2021). Social distancing measures, seen as a pivotal strategy in curbing the virus’s spread, led to a substantial reduction in outdoor activities, hindering physical exercise. Pregnant women, especially in high-prevalence areas, refrained from leaving their homes, even temporarily distancing from their spouses. The shutdown of transportation systems compounded challenges. Job layoffs surged, escalating unemployment rates and diminishing family incomes (Nowacka et al., 2021). Consequently, accessing antenatal care became increasingly inconvenient, reflecting the multifaceted impact of these measures (Bivia-Roig et al., 2020). However, this policy may have unintended consequences, such as reducing social support for pregnant women, including emotional support, appraisal support, informational support, and instrumental support (House, 1981), which may increase anxiety levels in pregnant women. A study conducted in Iran on 300 pregnant women who were not infected with COVID-19 found that 51.30% of them had a high level of anxiety related to the COVID-19 outbreak during their pregnancy (Mehdizadehkashi et al., 2021). A study conducted in the United States also found that pregnant women experienced high levels of anxiety during the COVID-19 outbreak and were more anxious about childbirth during the pandemic (Moyer et al., 2020). This is consistent with another study conducted in Thailand on 403 pregnant women during the first COVID-19 outbreak, which found that anxiety levels were significantly higher in pregnant women compared to those who were pregnant before the outbreak (Thongsomboon et al., 2020).

Based on the studies, social support is related to anxiety levels in pregnant women during the COVID-19 pandemic (Hocaoglu et al., 2020; Hamzehgardeshi et al., 2021). Moreover, it has been found that social support is negatively connected to anxiety levels in pregnant women during the COVID-19 pandemic (Azene et al., 2020; Kassaw and Pandey, 2020). This is significant given that COVID-19 is a new and rapidly spreading infectious disease, and information about the disease, particularly the COVID-19 vaccine, is not yet clear. Accessing and understanding accurate information for appropriate pregnancy care may be challenging. In Turkey, a study found that a higher level of health literacy about COVID-19 was significantly associated with lower levels of anxiety (*p* < 0.05) (Ugras et al., 2021).

The high level of anxiety may have an impact on pregnant women, pregnancy, and the fetus, such as vaginal bleeding, miscarriage, preterm delivery, and low birth weight (Hoyer et al., 2020; Nodoushan et al., 2020). Therefore, the researchers conducted a study to investigate the predictors of anxiety levels in pregnant women during the COVID -19 pandemic, in order to provide information for developing guidelines to reduce anxiety during the outbreak of COVID-19 or other similar emerging infectious diseases in the future.

## Objectives

1. To investigate anxiety levels among pregnant women during the outbreak of COVID-19.
2. To identify predictive factors for anxiety levels among pregnant women during the outbreak of COVID-19.
3. To conduct an in-depth exploration of social support, health literacy, and anxiety levels among pregnant women during the outbreak of COVID-19.

## Methods

The research conducted in this study included a mixed methods approach to investigate maternal anxiety and predictive factors of anxiety in pregnant women during the COVID-19 pandemic. The research proposal has been approved by Kuakarun Faculty of Nursing Institutional Review Board, Navamindradhiraj University, and Institutional Review Board Faculty of Medicine Vajira Hospital, as well as Bangkok Metropolitan Administration Human Research Ethics Committee.

### Population and sample group

The population in this study consists of pregnant women residing in Thailand, aged 18 years and older, in all stages of pregnancy, without any pregnancy complications, and with the ability to use a Google Form. The sample group for quantitative data collection includes pregnant women during the COVID-19 pandemic outbreak period. The sample size was determined using G*Power software, based on Cohen's (1992) calculation principle, with a power (1-β) of 0.95 and alpha value of 0.05. The sample group size was set to be 385 individuals, and an additional 10% were added to account by the researchers, resulting in a final sample group size of 424 individuals. Purposive sampling was used to select the sample group between November 2021 to May 2022, and the complete response rate was 404 individuals, or 95.28%.

### Measurements

Tools used for data collection include questionnaires which are divided into five parts as follows:

**Part 1:** Personal information includes age, education level, residential address, household income, employment status, pregnancy history, and complications during pregnancy.

**Part 2:** The measurement of health literacy about COVID-19 and pregnancy is adapted from the health knowledge and health behavior questionnaire for working-age population from the Department of Health Service Support, Ministry of Public Health (2020a). It is a Likert scale with 5 levels and consists of 24 items. The scores range from 24 to 120, and the scores are categorized according to Best's (1977) criteria. Scores between 24–56 indicate low knowledge, scores between 57–89 indicate moderate knowledge, and scores between 90–120 indicate high knowledge.

**Part 3:** The measurement of social support is an assessment of social support that the researchers developed based on the theoretical concept of social support by House (1981). It is a Likert scale with 5 levels and consists of 20 items. The scores range from 20 to 100 and are categorized according to Best’s (1977) criteria. Scores between 20–47 indicate low social support, scores between 48–75 indicate moderate social support, and scores between 76–100 indicate high social support.

**Part 4:** The measurement of anxiety related to COVID-19 infection and pregnancy: Data was collected using an adapted version of the Modified Pregnancy-Related Anxiety Scale (PRAS) (Moyer et al., 2020), which is a Likert scale with five levels and 15 items, scoring between 15 and 75 points. The scoring criteria for the modified PRAS was based on Best’s (1977) criteria, where scores between 15–35 indicate low anxiety, scores between 36–56 indicate moderate anxiety, and scores between 57–75 indicate high anxiety.

**Part 5:** In-depth interviews were conducted using open-ended questions created by the researchers. The interviews were conducted with 20 pregnant women, covering topics as following:

How do you feel anxious about COVID-19 infection and pregnancy?

What factors contribute to increasing your anxiety?

What factors contribute to reducing your anxiety?

The researchers conducted the quality testing of the questionnaire by having three experts to evaluate the content validity index (CVI) of the questionnaire. The CVI values for the health literacy about COVID-19 and pregnancy, social support and anxiety related to COVID-19 and pregnancy were 0.87, 0.80, and 0.87, respectively. Then, the revised questionnaire was tried out on a sample of 30 pregnant women with characteristics similar to the target population to assess the reliability using Cronbach’s alpha coefficient. The health literacy about COVID-19 and pregnancy, social support and anxiety related to COVID-19 and pregnancy of the questionnaire had Cronbach’s alpha coefficients of 0.96, 0.95, and 0.96, respectively.

### Data collection

The research was conducted in two phases as follows:

#### Phase 1: quantitative research

The objective of this phase was to investigate predictors of anxiety among pregnant women during the outbreak of the COVID-19 infection. The research was conducted using a questionnaire consisting of personal information, social support assessment, health literacy assessment, and anxiety assessment. The research project was promoted and distributed through an online platform, specifically a Facebook group comprising pregnant women, such as “Group of pregnant women talk” and “Group of newly mother talk”.

#### Phase 2: qualitative research

In this phase, in-depth interviews were conducted through video call to obtain rich and detailed data. The interviews were carried out using a set of open-ended questions developed by the researchers. The sample for this phase was selected through simple random sampling from volunteers who expressed their interest in Phase 1 to participate in Phase 2 of the research. The introduction and interview were made as scheduled, and participants were informed about the objective of the interviews conducted through VDO calls and were requested for audio recording consent. Once the participants agreed to take part in the research project, the interviews were conducted using the open-ended questions developed by the researchers. Each interview lasted between 15 to 20 min, and a total of 20 individuals were interviewed.

### Statistical analysis

Data analysis was conducted using packaged computer programs as follows:

1. Analysis of personal data, health literacy regarding COVID-19 and pregnancy, social support, and anxiety related to COVID-19 infection and pregnancy was performed using descriptive statistics, mean, and standard deviation.
2. Analysis of data from in-depth interviews was conducted using content analysis.
3. Analysis of personal factors, health literacy regarding COVID-19 and pregnancy and social support to predict the anxiety related to COVID-19 infection and pregnancy was performed using stepwise multiple regression analysis, with a significance level set at 0.05.

## Results

### Personal data

The data of the sample group consisting of 404 individuals was analyzed. The majority of the participants, 280 individuals (69.31%) were between the ages of 20 and 34, while 351 individuals (86.88%) were married. Regarding educational attainment, 132 individuals (32.67%) had completed junior high school. In terms of residency, 314 individuals (77.72%) lived in urban areas, and 224 individuals (55.46%) resided in areas at high risk of COVID-19 infection. In terms of monthly family income, 223 individuals (55.20%) reported an income range of 10,000 to 20,000 baht, and 193 individuals (47.77%) had stable employment and income. Furthermore, 201 individuals (49.75%) were in their third trimester of pregnancy (28–40 weeks). In terms of perceptions about the effectiveness of COVID-19 vaccines, 187 individuals (46.29%) believed that the vaccines offered moderate protection against COVID-19. A total of 253 individuals (62.62%) had previously been infected with COVID-19, and 331 individuals (81.93%) had close contacts who were infected. Additionally, 328 individuals (81.19%) had undergone quarantine for COVID-19 surveillance. The majority, 149 individuals (36.88%), had not yet received the COVID-19 vaccine.

### Anxiety related to COVID-19 infection and pregnancy

Anxiety related to COVID-19 infection and pregnancy was predominantly at a moderate level, ranging from 36 to 56 on the anxiety scale (mean = 47.78, SD = 11.49). Interviews revealed that pregnant women experienced anxiety in five areas: their own health status, the health of their fetus, the safety of their older child, the safety of their spouse, and the process of childbirth and hospital stay. The following quotes from pregnant women’s interviews illustrate their concerns:

“I worry about getting infected and concern about myself, the baby, and my eldest child, especially about my husband because he goes out the most.”

“I am afraid of getting infected because pregnant women have a weaker immune system and are more susceptible. I am afraid of giving birth without anyone to accompany because visitors are not allowed due to COVID-19. If I am away, there will not be anyone to take care of my older child.”

“I’m afraid of getting infected with COVID-19 before giving birth. What if something happens? I’m afraid I’ll have to undergo a surgery, and I worry if someone gets infected, they will have to undergoes surgery only. And I worry about giving birth method and they will not allow any relatives to accompany.”

In addition, interviews also revealed that factors contributing to increased anxiety among pregnant women include working suspension and loss of income, traveling outside, household members working or providing services outside, the need to interact with people, having pre-existing medical conditions, being in close proximity to individuals infected with the COVID-19 virus, and exposure to alarming news about the severity of the COVID-19 infection. The following are examples of interviews conducted with pregnant women:

“Watching news makes me more anxious. There are also reports of dying, even pregnant women. It make us scared, so we try not to watch the news too much.”

“The environment makes us more stressed. If people around us get infected, we become even more fearful. We do not know which places have the virus, and we are afraid of touching contaminated surfaces or accidentally open our masks.”

Factors that contribute to reducing anxiety include receiving support from family members, practicing strict personal protective to prevent infection, having a small number of family members, and residing in rural areas or non-high risk areas. The following are examples of interviews with pregnant women:

“When I’m not in the city and return to my hometown in the rubber trees plantation, I feel less stressed because I do not have to interact with people.”

“I’m glad to have my mother’s help. She buys food for me and assists me with financial matters when my resources are insufficient.”

### Health literacy regarding COVID-19 and pregnancy

Pregnant women have the health literacy related to the coronavirus disease 2019 (COVID-19) and pregnancy in a high level, ranging between 60 and 120 (mean = 96.36, SD = 14.23). From interviews, it was found that pregnant women seek information on their own and compare it with their friends or family members. They also consult healthcare professionals to confirm information at times. The following excerpts from interviews with pregnant women illustrate this:

“I search for knowledge on the internet because it’s easier. However, if something seems strange, I will ask the doctor when I visit the hospital because their explanation is more understandable and reliable.”

“I watch the news during dinner with my family. We discuss it together, and if there’s something I do not understand, I can ask others for answers. It makes me feel more at ease than being stressed by watching it alone.”

“At first, I was hesitant to get vaccinated because my siblings who gave birth earlier told me that breastfeeding might be affected. I was afraid I would not have enough breast milk for my baby. However, as I read information on pages, my understanding improved, so I decided to get vaccinated.”

### Social support

Pregnant women receive significant social support (mean = 83.99, SD = 11.34) reporting high levels of support. From interviews, it was found that pregnant women receive support from their spouses, parents, friends, colleagues, and medical personnel. This support encompasses various aspects, including childcare assistance, provision of food, financial aid, household items, information sharing, assistance with activities, and emotional encouragement, as the following interviews:

“A nurse from the public health service center came to my house, provided blood tonic, reminded me, and gave me encouragement. They advised me to take good care of myself during pregnancy so that the baby would be healthy. My partner also take cares of some heavy housework.”

“My partner goes out and buys things for us since I cannot go out. When we run out of essentials, my partner buys them, and the supplies last for about a week.”

“People around me try not to go outside for my sake. They help finding necessary items, while my mother prepares food for me. I bring food from home to eat during work hours. I do not buy and eat outside food. During lunchtime, I have a lunchbox at the office instead.”

Data of health literacy regarding COVID-19 and pregnancy, social support and anxiety related to COVID-19 infection and pregnancy are presented in Table 1.

**Table 1:** Health literacy regarding COVID-19 and pregnancy, social support and anxiety related to COVID-19 infection and pregnancy.

Data	Mean	S.D.	Level
Health Literacy regarding COVID-19 and pregnancy	96.36	14.23	High
Social support	83.99	11.34	High
Anxiety related to COVID-19 infection and pregnancy	47.78	11.49	Moderate

### Predicting factors of anxiety related to COVID-19 infection and pregnancy

From the preliminary test of agreement, it was found that the variables exhibited appropriate relationships for non-multicollinearity analysis based on the examination of Tolerance and VIF values, with Tolerance = 0.98 and VIF = 1.01, which passed the preliminary agreement. Considering the Durbin-Watson value, which is 1.83, it passed the preliminary agreement for non-autocorrelation. Multiple regression analysis using all three independent variables revealed that two of them significantly predicted anxiety related to COVID-19 and pregnancy at a statistically significant level of 0.05. Specifically, the health literacy regarding COVID-19 infection (HL) (*β* = 0.468) and social support (SS) (*β* = 0.283) together accounted for 32.80% of the variance in anxiety (*R*^2^ = 0.328). The results are presented in Table 2.

**Table 2:** Variable analysis of predicting factors of anxiety related to COVID-19 infection and pregnancy using stepwise multiple regression.

Predicting factors	*B*	S.E.	*β*	*t*	*p*-value
Health literacy regarding COVID-19 infection	0.378	0.033	0.468	11.365	0.000
Social support	0.287	0.042	0.283	6.875	0.000

Constant = 108.294; *R* = 0.573; *R*^2^ = 0.328; Adjusted *R*^2^ = 0.325; *F* 97.997; *p* < 0.05.

## Discussion

A majority of pregnant women experience moderate levels of anxiety, consistent with a study conducted by Ilska et al. (2022), which found that pregnant women during the COVID-19 pandemic had moderate levels of anxiety. This may be attributed to the government’s campaign to practice preventive measures based on the DMHTT principles. Statistical reports on the number of infections and deaths caused by COVID-19 have raised awareness among pregnant women about the importance of taking precautions. Healthcare services have implemented policies such as reducing hospital visits to minimize the infection, utilizing telemedicine, providing prenatal care through mail delivery of medications, and deploying volunteer healthcare workers for community support. Screening for COVID-19 risk factors for individuals entering communities has also been intensified, leading to moderate levels of anxiety among pregnant women.

However, interviews with pregnant women have revealed additional factors contributing to their anxiety. Firstly, the uncertainty of the disease as it is a newly emerged illness. Secondly, pregnant women express concerns about their family members, particularly their husbands who have to leave the house for work and manage various tasks on their behalf. Furthermore, they worry about their older children attending school and the potential for them to contract the virus and spread it to pregnant women or other family members. These concerns align with the previous studies (Yan et al., 2020; Ding et al., 2021; Wang et al., 2022), which have found that increased risk perception among pregnant women leads to greater self-protective behaviors. It is evident that pregnant women take more precautions during the pandemic compared to before their pregnancy, such as wearing double-layered face masks, frequently washing hands, and minimizing outdoor activities. Conversely, when pregnant women perceive a higher risk, it results in increased anxiety levels.

Furthermore, in the context of Thailand, pregnant women who experience pregnancy-related symptoms, upon arriving at the hospital, will undergo a COVID-19 screening. If there is a risk of infection, they will be isolated until test results come back negative. All pregnant women undergo COVID-19 testing, and if no infection is detected, they will be admitted to the labor room and receive regular medical care from doctors and nurses. However, for cases where COVID-19 infection is confirmed, pregnant women are required to stay in a separate, specifically designed negative-pressure delivery room. Husbands or relatives are not allowed to visit, and communication is facilitated through video call. Isolated care is provided by doctors and nurses. After delivery, the newborn is separated from the mother and kept in a separate room. In cases requiring cesarean section, disposable equipment is used, leading to increased expenses. These factors contribute to heightened anxiety levels among pregnant women. The research also found that the majority of the study sample resided in densely populated urban areas, where the prevalence of COVID-19 infections was higher compared to rural areas. This further increases the anxiety levels among pregnant women residing in urban areas, particularly in Bangkok, the capital city of Thailand, which has recorded more infections than any other province. These findings align with the study by Shangguan et al. (2021), which reported higher anxiety levels regarding COVID-19 infection among pregnant women residing in urban areas compared to rural areas.

According to the research findings, there is a high level of health literacy regarding COVID-19 infection and pregnancy. This knowledge can predict the level of anxiety experienced by pregnant women during the period of COVID-19 outbreaks. Pregnant women exhibit a high level of health literacy regarding COVID-19 infection and pregnancy because of easy access to various sources of information, predominantly through LINE and Facebook applications. In the context of Thailand, the general population has a 77.8% access rate to online social media (Kemp, 2022). Other sources of information include television programs and informative posters found in various locations, which are provided by government agencies in Thailand to disseminate diverse knowledge. The main methods for receiving news and information are through online platforms. Additionally, the government regularly presents news and guidelines on COVID-19 infection and practices through television broadcasts. However, some pregnant women report that excessive exposure to news has led to increased anxiety. This finding is consistent with the previous studies indicated that excessive news consumption can contribute to heightened anxiety (Wang et al., 2022). Health literacy is a crucial factor that enables pregnant women to stay well-informed and reduce their anxiety levels. The research findings demonstrate that knowledge regarding the health literacy of COVID-19 infection can predict the level of anxiety among pregnant women during periods of COVID-19 outbreaks. This is consistent with studies conducted in Japan and Vietnam, which found that pregnant women with higher health knowledge had lower levels of anxiety (Luong et al., 2021).

Furthermore, it has been found that social support for pregnant women during the COVID-19 pandemic is high. This is because the majority of Thai society consists of extended families, where family members have close relationships and a constant concern for each other. Additionally, pregnant women are viewed as individuals who should receive special care. In normal circumstances without disease outbreaks, family members often take good care of pregnant women as a norm. Moreover, Thailand’s healthcare system has a strong primary care system, with community health volunteers residing in the same communities as pregnant women, providing close care. This allows pregnant women to seek trusted advice and counseling. Consistent with previous research, it has been found that social support can predict the level of anxiety experienced by pregnant women during the COVID-19 pandemic (Khoury et al., 2021). Pregnant women in Thailand receive social support in four dimensions, as proposed by House's (1981) social support theory. These dimensions include emotional support, where family members provide encouragement, love, understanding, and reduce anxiety for pregnant women. Appraisal support involves receiving positive feedback from family members when pregnant women take good care of themselves. Informational support involves family members and friends assisting in finding news and information, aligning with interview data indicating that pregnant women have an interest in searching for information and have good access to information sources. However, when pregnant women receive a large amount of COVID-19 related information or news for an extended period, it can lead to increased anxiety. Pregnant women choose to receive information through family members or friends to help filter out violent content and provide practical guidance. Instrumental support involves mothers and husbands providing meals or purchasing food, as well as providing daily necessities and items for childbirth preparation. Additionally, healthcare personnel in the public health service deliver prenatal vitamins and other necessary medications to the homes of pregnant women or send them by mail. These factors contribute to the low levels of anxiety experienced by pregnant women in Thailand.

## Conclusion and implication

Pregnant women’s anxiety levels during the COVID-19 pandemic are at a moderate level. Predictive factors include health literacy and social support. Therefore, it is necessary to promote health knowledge among pregnant women, particularly through online sources, and provide support from family members and healthcare professionals. Furthermore, it is recommended to use health literacy and social support as guiding principles to develop a model of prenatal care for future implementation.

This study’s outcomes offer valuable insights applicable to prenatal care in high-prevalence areas of COVID-19 infection. The methodological rigor, exemplified by a robust sample size, enhances the study’s credibility. The use of standardized questionnaires assessing health literacy about COVID-19 and pregnancy, social support, and anxiety related to COVID-19 infection and pregnancy establishes a replicable framework for future research in analogous domains. The explicit delineation of tools and methodology contributes to the further scholarly utility of this study. The results of the study can be utilized in similar context countries.

### Limitations

This research exhibits some limitations that warrant consideration. Notably, the absence of a comparative analysis with non-pregnant cohorts restricts a comprehensive understanding of the broader population. The reliance on an online platform for data collection introduces a potential bias, as participants with greater data accessibility may possess elevated health literacy, potentially skewing concern levels downward. Moreover, the predominantly urban residence of volunteers may not be representative of pregnant women in rural settings lacking internet-connected mobile phones. These nuanced limitations underscore the need for cautious interpretation but also contribute to the study’s transparency.

### Suggestions

Subsequent investigations should adopt a comparative approach, evaluating the concerns of pregnant women against those of diverse population segments. Additionally, comparisons between urban and rural pregnant cohorts, as well as those with and without internet access, would offer valuable insights.

Furthermore, elevating health literacy on COVID-19 among pregnant women involves enhancing their online information access and critical analysis skills. Healthcare personnel should actively bolster social support by offering guidance to families caring for pregnant women and fostering clear communication to mitigate anxiety. The recruitment and training of Village Health Volunteers (VHV) is pivotal for localized care, especially during lockdowns, ensuring effective support for pregnant women.

## Data availability statement

The datasets presented in this study can be found in online repositories. The names of the repository/repositories and accession number(s) can be found in the article/supplementary material.

## Ethics statement

The studies involving humans were approved by 1. Kuakarun Faculty of Nursing Institutional Review Board, Navamindradhiraj University (KFN14/2021) 2. Institutional Review Board Faculty of Medicine Vajira Hospital (COA 152/2564) 3. Bangkok Metropolitan Administration Human Research Ethics Committee (E013h/64_EXP). The studies were conducted in accordance with the local legislation and institutional requirements. The participants provided their written informed consent to participate in this study.

## Author contributions

All authors listed have made a substantial, direct, and intellectual contribution to the work and approved it for publication.
